# Supplementary material for: Splice-Junction-Based Mapping of Alternative Isoforms in the Human Proteome
Source: Cell Rep. Author manuscript; Available in PMC 2020 Jan 15. (PMC6961840; doi:10.1016/j.celrep.2019.11.026)
Supplement: 2 [file NIHMS1546469-supplement-2.zip › DF1/PXD010154/Testis/Testis_18_PCBP2_SGLDASAQTTSHELTIPNDLIGCIIGR.pdf]

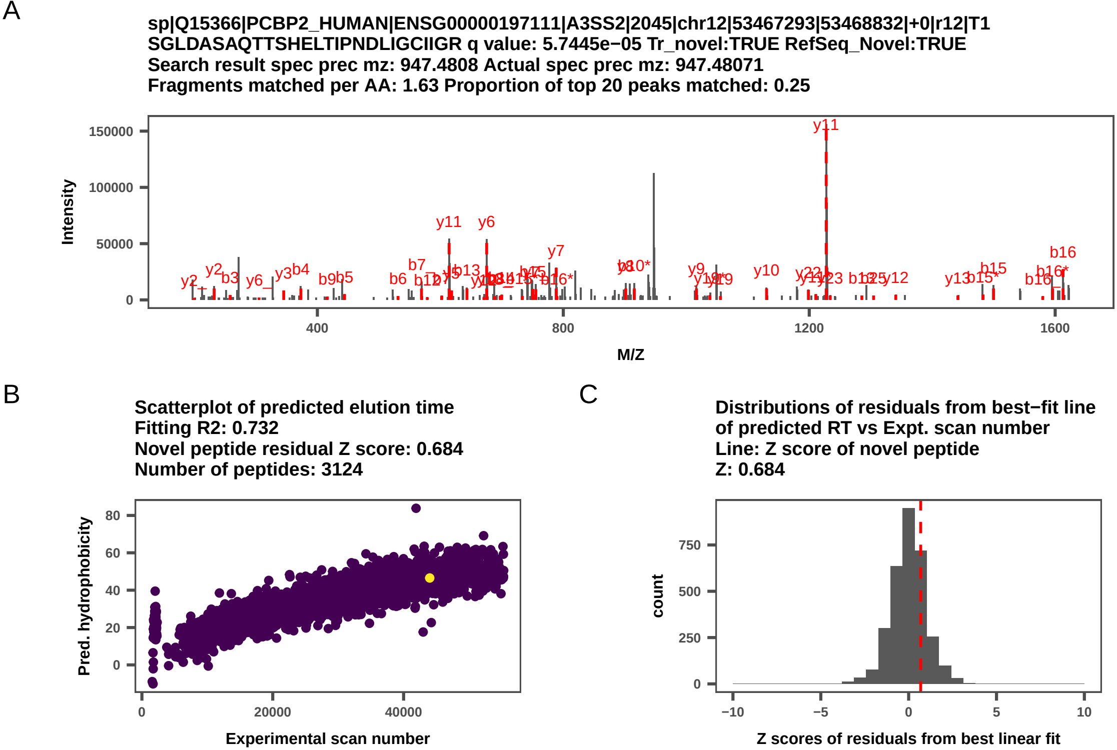
A
sp|Q15366|PCBP2_HUMAN|ENSG00000197111|A3SS2|2045|chr12|53467293|53468832|+0|r12|T1
SGLDASAQTTSHELTIPNDLIGCIIGR q value: 5.7445e−05 Tr_novel:TRUE RefSeq_Novel:TRUE
Search result spec prec mz: 947.4808 Actual spec prec mz: 947.48071
Fragments matched per AA: 1.63 Proportion of top 20 peaks matched: 0.25
Intensity
150000
100000
50000
0
400
800
1200
1600
M/Z
y11
y6
y7
y10
b16
B
Scatterplot of predicted elution time
Fitting R2: 0.732
Novel peptide residual Z score: 0.684
Number of peptides: 3124
Pred. hydrophobicity
80
60
40
20
0
0
20000
40000
Experimental scan number
C
Distributions of residuals from best−fit line
of predicted RT vs Expt. scan number
Line: Z score of novel peptide
Z: 0.684
count
750
500
250
0
−10
−5
0
5
10
Z scores of residuals from best linear fit
